# Strengthening Mental Health and Resilience Through Schools: Protocol for a Participatory Design Project

**DOI:** 10.2196/49670

**Published:** 2023-08-18

**Authors:** Jolan Kegelaers, Imke Baetens, Veerle Soyez, Martijn Van Heel, Lisa Van Hove, Paul Wylleman

**Affiliations:** 1 Brussels University Consultation Center, Faculty of Psychology and Educational Sciences Vrije Universiteit Brussel Brussels Belgium; 2 Sport Psychology and Mental Support, Faculty of Physical Education and Physiotherapy Vrije Universiteit Brussel Brussels Belgium; 3 Gezond Leven Brussels Belgium

**Keywords:** children, physical activity, psychological symptoms, sport, well-being, youth

## Abstract

**Background:**

Mental health problems are a main contributor to the global burden of disease in children and young people within urban environments. In response, the potential of both school- and sport-based mental health promotion interventions has been advocated. However, there exists limited insights into how sport-based interventions can be integrated within school environment. Moreover, there is a need to consider children and young people’s specific needs, challenges, and motivations when designing novel mental health promotion interventions.

**Objective:**

The Strengthening Mental Health and Resilience Through Schools (SMARTS) project aims to co-design an evidence-informed school-sport-based mental health promotion program. Specific objectives include (1) co-designing a multicomponent program, integrating sport sessions with class-based sessions, and complementing with educational modules for teachers and parents; (2) exploring how the mental health program can be implemented most effectively within the Brussels school system; and (3) conducting preliminary process and outcome testing of the program.

**Methods:**

A participatory design framework will be adopted to develop the program. This framework involves end users throughout the entire study process, from problem identification to intervention delivery and evaluation, while at the same time ensuring program development remains directly informed by the available scientific evidence.

**Results:**

Participant recruitment will commence in September 2023. The full project will be completed by March 2027.

**Conclusions:**

With this intervention, we aim to provide a direct contribution to the promotion of children and young people’s mental health within the Brussels school context. At a broader level, conducting and documenting this large participatory design project can, hopefully, inspire other researchers to tailor their mental health programs to specific populations.

**International Registered Report Identifier (IRRID):**

PRR1-10.2196/49670

## Introduction

### Overview

Mental health problems form one of the main contributors to the global burden of disease among young people. The ﻿first onset of most mental disorders typically occurs in childhood or early adolescence [1], with children and young people between the ages of 13 and 15 years being most at risk for first onset of psychological symptoms and distress. According to the World Health Organization [2], 1 in 7 children and young people is experiencing a mental disorder (eg, depression or anxiety), and suicide forms the fourth leading cause of death among this age group. Moreover, evidence suggests that up to 20% of school-going youth have engaged in nonsuicidal self-injury (NSSI) at least once [3]. NSSI forms a particularly relevant marker of mental health, as it is one of the strongest predictors of suicide [4] and is associated with a variety of other negative mental health outcomes in children and young people, including anxiety, depression, and personality disorders [5]. The mental health of children and young people also forms a particularly relevant and major social-urban challenge, as evidence suggests that mental health problems are more prevalent within metropolitan contexts compared to rural settings [6,7]. In Belgium, for example, inhabitants from the Brussels capital region tend to report significantly lower perceived well-being and higher prevalence rates of mental disorders, suicide attempts, and use of psychopharmacological medication compared to regional or national averages [8]. Moreover, the mental health of children and young people within urban settings has increasingly come to the forefront during the COVID-19 pandemic. Evidence suggests that the pandemic, as well as isolation and loneliness as a consequence of lockdown measures, have led to a considerable increase in symptoms of depression and anxiety in young people [9,10]. Even post the COVID-19 pandemic, the rates of psychological symptoms seem to remain remarkably high. Overall, the impact of mental health problems on young people is profound, as individuals experiencing psychological distress are particularly vulnerable to social exclusion, discrimination, stigma, educational difficulties, risk-taking behaviors, physical ill-health, and human rights violations [2].

These sobering facts highlight the need for evidence-informed mental health interventions for children and young people within urban settings. The World Health Organization [11] defines good mental health as "a state of well-being that enables people to cope with the stresses of life, to realize their abilities, to learn and work well, and to contribute to their communities. Mental health is an integral component of health and well-being and is more than the absence of mental disorders." This definition supports a 2-factor model of mental health [12], which distinguishes positive subjective well-being and psychopathology as 2 related but essentially distinct dimensions of mental health [11-14].

Concerning psychopathology, there is a clear need for preventive and early intervention programs to complement traditional clinical care. This is particularly important as many children and young people never seek out professional care [15]. In Belgium, for example, data indicate that up to 41% of adolescents who are experiencing severe mental health problems never receive professional treatment [8]. Moreover, even when professional treatment is reached, this typically does not occur until several years after the initial onset of mental disorders [1]. Finally, from a practical perspective, the increased recognition and prevalence of mental health problems in children and young people has severely strained the available health care system, leading to often month-long waiting lists for clinical mental health care. At the same time, the 2-factor model recognizes that mental health reflects more than merely the absence of mental disease or ill-health. Interventions may therefore equally consider a mental health promotion approach [12], focusing on children and young people’s ability to achieve developmentally appropriate tasks and enhance their self-esteem, mastery, resilience, social inclusion, and well-being [16,17]. As such, mental health promotion forms an “important component of the mental health intervention spectrum, which can serve as a foundation for the prevention and treatment of mental disorders” [16].

Existing mental health interventions for children and young people have commonly been delivered through school-based programs. School environments offer a unique opportunity to integrate mental health interventions within existing school structures, thereby reaching a far greater group of potentially at-risk children and young people compared to more traditional health care services [18]. In Flanders, for example, the government supports school-based health prevention primarily through activities that are rooted in the strategic plan “De Vlaming Leeft Gezonder in 2025.” The idea is to strive for an integrated policy (“Health in all policies”) that is reflected in important life domains (so-called “settings,” among other educational settings). A growing body of evidence indeed suggests that such school-based programs can be effective in promoting mental health and well-being [19-21], as well as preventing specific psychological symptoms such as NSSI and suicidality [22,23], although a better theoretical understanding of program outcomes and ways of sustaining them remains necessary. This is particularly important given recent suggestions that, in certain cases, universal school-based interventions may not only be ineffective in promoting mental health but even actively cause harm to children and young people [24].

In addition to school-based initiatives, there has been increasing interest in the potential of sport and physical activity to promote mental health in young people. There exists strong evidence indicating the positive effects of physical activity in and of itself on the mental health and well-being of children and young people [25,26]. When used purposefully and deliberately, sport can also form an important vehicle for positive youth development [27]. The notion of positive youth development closely connects with mental health promotion, as it reflects a strength-based approach aimed at providing optimal developmental experiences to enable individuals to lead healthy, satisfying, and productive lives, both as a youth and later in life [28]. Additionally, sport can provide a “hook” to grab children and young people who would otherwise not find their way to traditional help-seeking pathways (eg, men and people of minority ethnic backgrounds) [29]. In particular within major urban areas, sport has the potential to reach and engage underprivileged, difficult-to-reach and at-risk populations, and has been posited as a cornerstone for inclusive and comprehensive social and well-being policies [30].

Despite the potential of sport-based interventions, there still exists a lack of research into the use of sport as a vehicle to promote mental health in young people, and existing initiatives often lack a clear theoretical or evidence base [31]. Moreover, existing programs have mostly been limited to organized sport contexts. To date, only a single study has developed a school-based mental health program that aims to integrate and complement physical activity sessions with traditional classroom-based sessions [32]. Although insightful, this intervention was developed in the United Kingdom as a response to specific challenges in their local communities. As such, it is unclear to what extent this intervention is transferable to the Brussels context. Moreover, the intervention specifically targeted pupils with a diagnosed mental illness rather than providing a broad mental health promotion intervention targeting the entire pupil population [32].

In sum, there is a timely and important need to develop and test novel evidence-informed mental health promotion programs for children and young people within the Brussels urban setting. The context of Brussels is unique as it is highly polarized geographically, economically, and socially, with a large youth population of minority ethnic background and lower socioeconomic status [33]. These factors have been associated with increased mental health stigma and form crucial barriers toward seeking and accessing traditional forms of mental health care [34]. To address such issues, a mental health promotion program integrating class-based sessions with sport sessions may offer considerable potential. To maximize impact, such a program would be best designed as a multicomponent intervention [18,19], offering multiple intervention components targeting both the promotion of subjective well-being (eg, strengthening resilience and promoting mental health literacy [MHL]) and the prevention of key psychological symptoms (eg, NSSI prevention) [12]. Moreover, interventions may also benefit from adopting a multilevel approach, by engaging multiple stakeholders (eg, parents and teachers) around the individual [2,18]. Such a holistic “whole-school” approach focuses on leveraging social and environmental influences to promote positive mental health in adolescents. At the same time, to be effective and sustainable, interventions need to account for the complexity of each target context and carefully tailor intervention components to the needs, challenges, motivations, and key competencies of the program’s end users [18].

### Moving Toward a Citizen Science Approach

Although school-based mental health programs have been around for over 3 decades, the majority of these interventions have been developed in the United States. In their review of the literature, Kuosmanen et al [18] noted that interventions tend to show less consistent positive results when they are directly transferred to other countries or contexts. Both cultural and contextual factors play a crucial role in determining a program’s impact, and one may, therefore, question to which end existing programs are applicable to handle specific local challenges [18]. Moreover, there seems to exist a “science-to-practice gap” in implementing such preventive mental health interventions [18]. Despite promising results, many large-scale randomized controlled trials of school-based mental health programs have proven difficult to sustain and upscale within “real-world” settings [20] and scholars have noted high levels of attrition within such programs [21]. All in all, these issues highlight the need for interventions to be carefully designed in function of the needs and challenges of the end users within their specific cultural and geographical context and, at the same time, be engaging and motivating to ensure end users are willing to start (and continue participating in) the program. In other words, there is a need to move from discrete intervention packages to creating sustainable implementation structures and climates [18].

Citizen science, or more specifically, a participatory design approach, might, therefore, be particularly suited to tailor a mental health program to the specific needs, challenges, and motivations of children and young people within the Brussels urban context. Participatory design “offers an evolving set of critical, conceptual, and practical tools to support the active participation of users in the design of different systems, services, and products” [35]. As such, interventions are designed from the perspective of the end user and actively involve them as co-designers. Such participatory research, promoting community participation, is particularly important to address “wicked problems,” wherein different stakeholders may hold disagreements regarding the interpretation of the problem and the science behind it, as well as conflicting values, goals, and life experiences [36]. As such, addressing these problems cannot be achieved by relying solely on interdisciplinary, expert-driven approaches. Concerning the design of mental health programs, participatory design forms an active affirmation of young people’s rights to define their own mental health goals and be involved in their own mental health care [35]. Benefits of adopting a participatory design approach toward developing a mental health program can include addressing stigma in intervention end users, involving populations who are otherwise hard to reach within traditional mental health care (eg, young men with minority ethnic background), creating a “shared language” between researchers and participants, and ultimately improving the likelihood of the program being used in the applied field [35]. Participatory design is also particularly suited to develop an intervention that is evidence-informed and, at the same time, engaging for children and young people. Similar participatory design approaches are currently being implemented to develop evidence-informed mental health interventions in other populations, including sexual and gender minority youth [37] and young people with schizophrenia [38].

### Research Objectives

Given the state-of-the-art presented above, the current Strengthening Mental Health and Resilience Through Schools (SMARTS) project aims to co-design a comprehensive, novel, evidence-informed school-sport-based mental health promotion program, developed with, and tailored for children and young people (aged 13-15 years) within the Brussels urban context. The specific objectives set out in this project include (1) co-designing a multicomponent (ie, MHL, strengthening resilience, prevention of NSSI and suicidality, and other psychological symptoms) mental health program for pupils, integrating sport sessions with class-based sessions, and complemented with educational modules for teachers and parents; (2) exploring how the mental health program can be implemented most effectively within the Brussels school system; and (3) conducting preliminary process and outcome testing of the entire mental health program.

To achieve these objectives, a participatory design approach will be central to the project. This participatory design will be informed by the framework set out by Hagen et al [35]. The central principles within this guiding framework are (1) the involvement of young people as active participants throughout the entire research process; (2) the active contribution of young people in idea generation and program design; and (3) the continuous evaluation of the program from the perspective of young people in terms of relevance, meaningfulness, and engagement.

### Underpinning Intervention Principles

The SMARTS intervention components will be informed by several underpinning, evidence-informed principles, and theories. These principles will provide structure and help shape the participatory design process.

#### MHL Framework

MHL is defined as “understanding how to obtain and maintain positive mental health, understanding mental disorders and their treatments, decreasing stigma related to mental disorders, and enhancing help-seeking efficacy” [39]. It includes knowledge of preventive strategies, the ability to recognize warning signals for mental health problems, awareness of help-seeking options, and first aid skills to help others who are struggling with mental health problems. A lack of MHL has been associated with increased stigma and poor symptom recognition and serves as a barrier toward help-seeking behaviors [15]. As such, developing MHL in young people and their main “helpers” (eg, parents and teachers) has been recognized as a key component for preventive and early intervention programs [40].

#### Psychological Resilience

Resilience is commonly defined as the capacity to withstand or quickly recover from stressors [41]. It entails a dynamic process of adaptation wherein individuals learn to draw on their personal and environmental resources to maintain mental health under stressful life circumstances. Within the SMARTS project, the focus on resilience reflects a strength-based approach with an emphasis on stress and emotion regulation skills as well as the capacity to build and draw upon social support networks [42].

#### Onset Prevention of Psychological Symptoms

Promoting MHL and resilience may also be complemented with interventions aimed at preventing the onset of psychological symptoms, such as NSSI. A meta-analysis by Fox et al [43] has demonstrated the insignificant effects of interventions that focus on reducing or preventing NSSI as a stand-alone intervention. However, a recent pilot study investigated the differences between a general in-classroom mental health prevention program called “Happyles” and a school-based prevention program called “HappylesPLUS” which also targeted NSSI by adding a 1-hour NSSI-focused psychoeducation module [23]. The extra module focuses on key variables that impact the onset of mental health problems in general and NSSI specifically, such as the impact of social media on mental health, negative body images, and peer stigmatization. Both groups reported a reduced likelihood of engaging in NSSI in the future 6 weeks after receiving the program in comparison to before receiving the program. Results also indicated increased emotional awareness in both groups. However, analysis of the qualitative data led to the indication that “HappylesPlus” may have direct benefits for some students with lived experience (eg, an increase in help-seeking behavior for NSSI) [23]. Building on this pilot project, the SMARTS project will equally include an NSSI-focused psychoeducational module within the intervention and will broaden its scope to include other prevalent mental health problems in the Brussels region, such as suicidality and addiction.

#### Sport-for-Development

Sport is often considered a vehicle for the promotion of desirable social and psychological outcomes. For example, with regard to mental health, sport can be a context to promote both MHL and resilience [29,44]. However, sport-for-development scholars have noted that beliefs surrounding the developmental potential of sport in and of itself are often naive, idealistic, and based on limited empirical support [45]. It is crucial to carefully consider the context and conditions under which sport might contribute to such desirable psychological outcomes. Within this project, we therefore focus on a sport plus approach, defined as interventions “in which sports are adapted and often augmented with parallel programs in order to maximize their potential to achieve developmental objectives” [45]. Within such a sport-plus approach, we will critically examine how ﻿sports activities can be shaped, adapted, and augmented to maximize the potential to achieve the envisioned outcomes, rather than solely relying on the supposed direct impact of sport and physical activity in and of themselves on children and young people’s mental health [25,26].

## Methods

### Overview

As stated, this study will use the participatory design framework presented by Hagen et al [35], as illustrated in Table 1. This framework identifies 6 research stages that provide a structured approach to involving end users throughout the entire study process, from problem identification to intervention delivery and evaluation, while at the same time ensuring program development remains directly informed by the available scientific evidence.

**Table 1:** Overview of the study stages.

	WP 1^a^			WP 2^b^			WP 3^c^		WP 4^d^
	IDENTIFY	DEFINE	POSITION		CONCEPT	CREATE		USE	
Aim of the study stage	Identify the problem from the perspective of the end users	Define the problem space and objectives within them	Understand how the intervention needs to be positioned in order to have the envisioned impact		Identify, generate, and evaluate concepts that represent what the intervention needs	Evolve, build, and refine an intervention that is useful and usable		Deliver, use, and evaluate the intervention based on the experiences of the end users	
Methods to enable active participant involvement	WP 1 focus group interviews with pupils, teachers, and parents	WP 1 focus group interviews with pupils, teachers, and parents	WP 2 co-design workshops with pupils, teachers, and parents		WP 2 co-design workshops with pupils, teachers, and parents	WP 3 pilot study with detailed process evaluation by intervention end users		WP 4 implementation and detailed process and outcome evaluation by intervention end users	
Evidence-informed research activities	Consultations with experts and review of epidemiological data	Scoping review of risk factors and resources for adolescent mental health	Consultations with experts and review of behavior change theories underpinning mental health interventions		Consultations with experts to establish relevant process, impact, and outcome indicators	Build and test (qualitative and quantitative) data collection battery		Group-controlled quasi-experimental study	

^a^WP 1: work package 1.

^b^WP 2: work package 2.

^c^WP 3: work package 3.

^d^WP 4: work package 4.

### Participants

#### Pupils

For the first 4 research stages (IDENTIFY, DEFINE, POSITION, and CONCEPT) we will recruit approximately 60 high-school pupils enrolled within the Brussels school context (aged 12-15 years). Within this sample, we will strive for a relatively heterogeneous group, recruiting participants from different schools, different education levels, different ethnic backgrounds, and with a balanced gender distribution. Within the CREATE research stage, we will sample an additional 40 pupils, representing 2 different classes. These classes will be purposefully selected from different schools to complete a pilot study of the proposed intervention. Finally, for the final research stage (USE), 300 participants will be recruited to complete the intervention, with another 300 pupils acting as matched controls. Pupils will be recruited from different schools, different educational levels, and different ethnic and socioeconomic backgrounds. The total number of participants within the USE phase is sufficient to ensure enough power for the planned statistical analyses and takes into account an attrition rate of 30%.

#### Teachers

For the first 4 research stages (IDENTIFY, DEFINE, POSITION, and CONCEPT), we will recruit approximately 24 high-school teachers. Within this sample, we will purposefully sample those teachers who will be involved in later stages of the delivery of the intervention. Specific attention will also be paid to involve physical education teachers. Within the CREATE research stage, we will sample 15 teachers to participate in the pilot study, representing 2 different schools. Finally, for the final research stage (USE) 50 teachers will be recruited from different schools to participate in the educational module for teachers.

#### Parents

For the first 4 research stages (IDENTIFY, DEFINE, POSITION, and CONCEPT) we will recruit approximately 24 parents of children enrolled within the Brussels school context. Within this sample, we will strive for a relatively heterogeneous group, recruiting parents of children from different schools, different education levels, different ethnic backgrounds, and with a balanced gender distribution. Within the CREATE research stage, we will sample 15 parents to participate in the pilot study, representing parents of pupils from 2 different schools. Finally, for the final research stage (USE) 50 parents will be recruited from different schools to participate in the educational module for parents.

#### Experts

For the IDENTIFY, POSITION, and CONCEPT research stages, an interdisciplinary group of applied and academic experts will be involved to help shape the evidence-informed intervention. We expect approximately 16 experts to be involved within the project’s life span. These experts will be recruited based on their specific expertise and applied experience within the project topic and setting and through the different project partners. These experts will consist of the following:

1. 4 school directors and 4 “zorgcoördinators” (care coordinators) from different schools within the Brussels region;
2. 2 school counseling consultants from different schools within the Brussels region;
3. 2 government prevention advisors with specific expertise in mental health and well-being;
4. 4 academic experts with specific expertise in mental health and well-being and (clinical) sport psychology.

### Procedures

Based on the project design outlined above (see Table 1), 4 main work packages are proposed. Each of the work packages is described separately below.

#### Work Package 1

The first work package will encompass the IDENTIFY and DEFINE research stages. Key activities and milestones within this work package include the following:

1. Focus group interviews with pupils, teachers, and parents: focus groups will be conducted to (1) understand the problem from the perspective of the end users and (2) help define objectives to address this given problem.
2. Consultations with experts: consultations will be conducted to understand the problem from the perspective of applied and academic experts.
3. Scoping review of the literature: a scoping review will be conducted to (1) evaluate epidemiological evidence and (2) identify risk factors and resources for adolescent mental health.

#### Work Package 2

The second work package will encompass the POSITION and CONCEPT research stages. Key activities and milestones within this work package include the following:

1. Co-design workshops with pupils, teachers, and parents: co-design workshops will be conducted to understand how the intervention needs to be positioned from the perspective of the end user and generate intervention content.
2. Review of behavior change theories: a brief literature review will be conducted on leading behavior change theories that can aid in the design of engaging, evidence-informed intervention content.
3. Consultations with experts: consultations with experts will be conducted to establish key indicators to evaluate the intervention process, impact, and outcome based on content developed during the co-design workshops.

#### Work Package 3

The third work package will encompass the CREATE research stage. Key activities and milestones within this work package include the following:

1. Pilot study with detailed process evaluation by intervention end users; a detailed pilot study will be conducted to test the intervention with a small sample of end users.
2. Build, test, and finalize data collection battery (quantitative and qualitative); a battery will be developed and tested during the pilot study based on the established indicators for intervention process, impact, and outcome (see WP 4).

#### Work Package 4

The fourth work package will encompass the USE research stage. Key activities and milestones within this work package include the following:

1. Implementation of a group-controlled quasi-experimental intervention: a quasi-experimental intervention will be conducted, randomly allocating participants at group level (ie, class) to either experimental or control conditions. The experimental condition will receive the intervention over a 6-month period, whereas the control group will serve as a “usual care” control group. Additionally, a selected sample of teachers and parents of pupils within the experimental group will be provided with the complementary educational modules.
2. Pretest, posttest, and 3-month follow-up (quantitative and qualitative) evaluations; a 3-wave data collection design will be adopted to collect both qualitative and quantitative data on the intervention process, impact, and outcome. These include pretest, posttest, and 3-month follow-up data collection points.

### Data Collection

Throughout the project, a mixed methods approach to data collection will be adopted. Qualitative data will be collected primarily through focus group interviews and co-design workshops. Each focus group and co-design workshop will be both audio- and video-recorded. Audio recordings will be transcribed verbatim, and transcriptions will be used for further analysis. Video recordings can be used to clarify the context of certain statements or to ascertain group dynamics within the interviews or workshops that cannot be discerned through audio alone.

Quantitative data will also be collected to evaluate program outcomes during the CREATE and USE research stages. In line with our participatory design approach, the determination of the most relevant project outcomes and the development of a corresponding test battery to assess these outcomes will be based on a co-design process during the project life span. As such, we have purposefully not determined an a priori set of outcome measures. However, an indicative set of outcome measures includes the following:

1. subjective well-being, as measured using the World Health Organization-Five Well-Being Index (WHO-5) [46];
2. psychological distress, as assessed using the Kessler Psychological Distress Scale [47];
3. mental health stigma, as assessed using the Peer Mental Health Stigma Scale [48];
4. help-seeking intentions, as measured by the ﻿Attitudes Toward Seeking Professional Psychological Help Scale-Short Form [49]; and
5. emotion regulations, as measured by the ﻿Difficulties in Emotion Regulation Scale [50].

### Data Analysis

Qualitative data will primarily be analyzed using reflexive thematic analysis. Reflexive thematic analysis is particularly suited to gain sense and meaning from participants’ lived experiences while at the same time recognizing the subjectivity of human experience and emphasizing that meaning-making from this experience is the result of a process of cocreation between the participant and the researcher [51]. To support our co-design principles and involve participants as active contributors throughout all phases of the analysis process, member reflections will also be used. Member reflections are structured opportunities for participants to provide feedback and reflection on tentative qualitative analysis, not as a way to verify the truthfulness of the analysis but rather as a way to generate novel insights and data [52].

Within the USE research stage, analysis of the main outcome measures will be calculated using a mixed-model ANOVA including 2 groups (mental health program vs control group) and measures at 3 time points (pre, post, and 3-month follow-up). Missing data will be dealt with by using appropriate methods.

### Ethical Considerations

Institutional ethics approval has been requested and will be obtained for the SMARTS project. All participants will provide active, informed consent before their participation. For the participating children and young people, informed consent will also be obtained from a parent or legal guardian. The informed consent forms and participant information sheets are currently also under review by the institutional ethics board. Given the sensitive nature of the topic, the information sheets will include contact details from the researcher as well as several currently available mental health resources, should the participants feel the need for support following their participation in the study. All data collected throughout the project will be pseudonymized and stored for a duration of 10 years within a secure data repository following the completion of the project.

## Results

Funding for the SMARTS project was approved in January 2023. Upon obtaining ethics approval, participant recruitment for the first research phase will commence in September 2023. The full project will be completed by March 2027.

## Discussion

Children and young people living within urban environments form a particularly at-risk population for mental health problems [2]. School-based mental health programs have, therefore, often been proposed as key interventions to target this population [19-21]. Additionally, sports have also been proposed as a context for mental health promotion programs, as well as a “hook” to attract children and young people to such programs [32]. Nevertheless, there currently exists a “science-to-practice gap” in the implementation of mental health promotion programs for children and young people [18], with existing interventions often proving difficult to sustain and upscale to broader settings. One potential solution to this issue might lie in the use of participatory design approaches, focusing on the active involvement of intervention end users (ie, children and young people) as co-designers throughout all phases of intervention development. The SMARTS project, thus, aims to develop an intervention that is evidence-informed and, at the same time, engaging for children and young people, through the adoption of a rigorous participatory design approach [35].

To achieve this objective, the SMARTS project will co-design and evaluate a comprehensive evidence-informed school-based mental health promotion program, integrating sport sessions with class-based sessions, complemented with educational modules for teachers and parents. This intervention is targeted at school-going children and young people aged 13-15 years. This particular age range was chosen as it coincides with the typical age during which children and young people are at risk for first onset of psychological symptoms and distress [1]. The mental health promotion program will integrate principles based on MHL [39], psychological resilience [41], existing school-based programs to prevent psychological symptoms [23], and insights from the sport-for-development literature [45]. To develop the program, the rigorous participatory design framework presented by Hagen et al [35] will be adopted. Through several consecutive research stages, the SMARTS project will (1) co-design the multicomponent program, (2) explore how the intervention can be implemented most effectively within the Brussels school system, and (3) conduct preliminary process and outcome testing of the full mental health promotion program. A key challenge when conducting such a participatory design project lies in the recruitment and activation of all relevant stakeholders within the co-design process. However, this can be mitigated as the SMARTS consortium, which includes local school organizations, will play an active role in both participant recruitment and the design and implementation of the eventual organization.

With this intervention, we aim to provide a direct contribution to the promotion of children and young people’s mental health within the Brussels school context. This is particularly relevant as children and young people within the Brussels urban context tend to report higher prevalence rates of mental disorders, compared to regional or national averages [8]. In part, this may be attributed to increased mental health stigma, as well as experienced social and economic barriers toward traditional mental health care. At a broader level, we hope that conducting and documenting large participatory design projects like SMARTS can inspire and guide other researchers to tailor mental health interventions to specific at-risk populations.

## Appendix

Multimedia Appendix 1 Peer-review report from VUB Research (Brussels, Belgium).

## Abbreviations

MHL: mental health literacy
NSSI: nonsuicidal self-injury
SMARTS: Strengthening Mental Health and Resilience Through Schools

## Data Availability

Data gathered during the SMARTS project will be stored in a restricted-access data repository. These can be made available upon reasonable request to the authors.
