# Supplementary material for: Strengthening Mental Health and Resilience Through Schools: Protocol for a Participatory Design Project
Source: JMIR Res Protoc. 2023 Aug 18;12:e49670. doi: 10.2196/49670 (PMC10474515; doi:10.2196/49670)
Supplement: Multimedia Appendix 1 [file resprot_v12i1e49670_app1.pdf]

VUB CITIZEN SCIENCE PROJECT CALL
Jury Feedback

Project name: SMARTS
Applicant: Imke Baetens

General remarks

Juror 1: This is a very well considered and currently important project that could help many young people with their mental health issues, directly and especially indirectly through application of learnings in the future. The consortium has been well put together with appropriate societal partners, and researchers' experience in the field is considerable.

Juror 2: Overall this is an improtant area of research that has the potential for significant impact. The citizen science approach proposed is a clear strength of the research in that it effectively balances scientific expertise with end-user needs and preferences. The methodogies employed in this study and its outcomes will be of interest to the scientific community.

Juror 3: My congratulations, the methodology in this proposal very well described and highly relevant for what is a clear and urgent need, the Brussels context is relevant but not central to its success, so a tiny bit off the bulls-eye, and I do think you'll face some recruitment challenges - but given time and attention I don't think this will be a significant hurdle and is not othewise a flaw in the proposal

Excerpts from jury reports

Scientific Merit

Juror 1: Objectives are clear and relevant, well argued. The approach and methodology is well outlined, appropriate for the proposed study and interventions. Consideration of participant motivation and actual recruitment is missing though. The study could lead to new insights and applicable interventions in the mental health domain which are needed.

Juror 2: The current proposal targets youth mental health, a topic of significant relevance and importance both in Brussels and globally and aligns with the Sustainable Development Goal of promoting good health and well-being in youth. The applicants provide a clear case to develop and test novel preventive evidence-informed mental health interventions for adolescents and the need for a multicomponent and multi-level intervention that targets both the promotion of wellbeing and the prevention of key psychological symptoms, such as non-suicidal self-injury.

The aims of the research were clearly defined and included the co-design and preliminary evaluation of a school-based intervention and the identification of implementation strategies to

ensure intervention longevity. The inclusion of parent and teacher modules in the intervention, to build the support network, is a clear strength of the proposed intervention.

In line with the citizen science approach, a Participatory Design (PD) framework was proposed to form the basis of the proposal and consists of six research stages that will actively involve young people, parents, teachers throughout the research process, including in idea generation, programme design and evaluation, while also being informed by experts and the available scientific evidence. This proposed approach is a real strength of the application, as it uniquely balances academic evidence and knowledge with end-user expertise, preferences and expectations.

The proposed methods within the six research stages were generally well described and will enable the research objectives to be met. The design and analysis of the focus groups and workshops was clear, and the anticipated outcomes measures for the final evaluation appear appropriate (noting that they are not finalised given the planned co-design approach).

I did have some questions about the final evaluation phase that I thought the applicants could have provided more detail on. For instance, did they account for the clustering (randomization by class) in the sample size calculation for the evaluation? This requires calculation of the design effect based on the intraclass correlation coefficient (ICC). The expected effect size of the intervention was also not provided or justified. Relatedly, it did not appear that the clustering was accounted for in the proposed analyses and it was unclear why the applicants were proposing a MANOVA and not a mixed model repeated measures ANOVA that would allow for clustering and account for missing data.

Lastly, I would have liked to see a little more information on the proposed intervention, acknowledging that it is intended to be co-developed. For example, some further detail on anticipated length, format and content (which was alluded to) for the student intervention, as well as the parent and teacher modules, would have further strengthened the application.

Impact

Juror 1: The potential impact on society is large, as youth mental health is a pertinent and current issue. New applicable knowledge will be created in this field, leading to the potential impact on society. Target groups are clearly identified. The social benefit is potentially large for participants in the study, though the controls would not benefit - this could be mitigated with a follow-up intervention for those students. There is no real social element set out in the study, though 'buddy-groups' or similar could have been part of the planning to include also this element.

Re. scientific dissemination: only 1 publication is planned as Open Access - ideally all would be so. No mention of other avenues, e.g. conferences, symposia, etc.

Other dissemination: policy makers are mentioned specifically, though have at no time during the project been consulted - this is a shortcoming. The is no outlined dissemination to project participants, or even mention of project partners and teachers as important audiences for dissemination - this is a major shortcoming. Also, dissemination is only planned for the last 3 quarters of the project - another shortcoming. The project will strengthen the VUB network, though there is no mention of this.

Juror 2: The current proposal has the potential for significant social and academic impact, including the prevention of mental health problems in young people and evidence for the effectiveness of a new school-based interventions that could be applied in other countries. The proposed intervention is somewhat novel in its targeting of a range of psychological and wellbeing factors and the inclusion of parent and teacher modules. The targets of the intervention were clear and all end users (young people, parents and teachers) were actively involved in the research process. This is a clear strength of the application, as the resulting intervention will likely be more engaging to the population and meet their needs. If found to be effective, the new intervention could have very positive social benefits for young people with reductions in mental health symptoms and promotion of wellbeing and resilience. The citizen science approach proposed by the applicants was comprehensive and would further strengthen the work of the VUB in this area. The proposed dissemination strategies were clear and would ensure outputs were accessible to both academic and non-academic audiences.

Implementation of the project plan

Juror 1: Deliverables and milestones are not clearly outlined, but can be deduced from the WP descriptions. As such, they are relevant and realistic.

The project design is appropriate for the time given. Staff identified are relevant with the necessary expertise to see the project through successfully.

Partners have signed LoI, though no costs are outlined in those letters.

Results are not planned as Open Access, not even all publications which is a major shortcoming. No further details on data, etc. but as data are sensitive, there may be limits to how open they can or should be - no discussion of this. IRB approval will be sought.

The budget is well argued, though there are no quotes.

Juror 2: The research team appear to have the skills and expertise to successfully execute the proposed program of research and will be well supported by a strong partnership team who are invested in the proposed research both in terms of the need for preventative approaches to address limited availability of clinical services and the clear application of the research beyond the research period. All partners expressed a commitment to applying the research beyond the research period. The project milestones generally appear appropriate although the feasibility of th,e project would be strengthened with the inclusion of additional paid staff, particularly given the number of research activities being undertaken and the need to develop a new intervention. The budget was well justified but didn't appear to include intervention development costs (such as delivery manuals, resources, activities). It was not clear how these would be covered.

Juror 3: I'm concerned that the project is not adequately staffed, the planning and effort involved in the communication tasks seems under-estimated to me, but this might be sufficiently addressed by the roles of Gezond Leven and SGR.

Implementation of the citizen science approach

Juror 1: Citizens are planned to be actively involved throughout the project - this is very good. Target groups have been identified and activities are clearly outlined and suitable for the target groups. This project could not be completed without the direct involvement of citizens.

The recruitment and selection procedures are lacking in details and there is no consideration of participant motivations for involvement or discussion of benefits or incentives for participants. Recruitment may prove more difficult than anticipated.

The outlined workshops with students, parents and teachers would benefit from more consideration. It is not clear from the description whether these will be mixed groups or separate target groups alone. From the young people's perspective, separate groups would be beneficial to ensure they open up to researchers (and don't feel awkward with parents or teachers present, even if they do not know the parents or teachers).

Ethics and privacy have not been described, though from the experience of the researchers, it is anticipated that these aspects will be approriately addressed.
